# Quality of Care of Hospitalized Internal Medicine Patients Bedspaced to Non-Internal Medicine Inpatient Units

**DOI:** 10.1371/journal.pone.0106763

**Published:** 2014-09-03

**Authors:** Jessica Liu, Joshua Griesman, Rosane Nisenbaum, Chaim M. Bell

**Affiliations:** 1 Department of Medicine, University Health Network, Toronto, Ontario, Canada; 2 Royal College of Surgeons in Ireland, Dublin, Ireland; 3 Li Ka Shing Knowledge Institute, St. Michael's Hospital, Toronto, Ontario, Canada, Dalla Lana School of Public Health, University of Toronto, Toronto, Ontario, Canada; 4 University of Toronto, Department of Medicine, Toronto, Ontario, Canada; 5 Institute for Health Policy Management and Evaluation, University of Toronto, Toronto, Ontario, Canada; 6 Department of Medicine, Mount Sinai Hospital, Toronto, Ontario, Canada; University of Pittsburgh Medical Center, United States of America

## Abstract

**Background:**

When the number of patients requiring hospital admission exceeds the number of available department-allotted beds, patients are often placed on a different specialty's inpatient ward, a practice known as “bedspacing”. Whether bedspacing affects quality of patient care has not been previously studied.

**Methods:**

We reviewed consecutive general internal medicine (GIM) admissions for congestive heart failure (CHF), chronic obstructive pulmonary disease (COPD), and pneumonia at St. Michael's Hospital in Toronto, Canada, from 2007 to 2011 and examined whether quality of care differs between bedspaced and nonbedspaced patients. We matched each bedspaced patient with a GIM ward patient admitted on the same call shift with the same diagnosis. The primary outcome was the ratio of the actual to the estimated length of stay (ELOS). General and disease specific measures for CHF, COPD, and pneumonia (e.g. fluid restriction) were evaluated, as well as 30-day Emergency Department (ED) and hospital readmissions.

**Results:**

Overall, 1639 consecutive admissions were reviewed, and 39 matched pairs for CHF, COPD and pneumonia were studied. Differences in both general and disease specific care measures were not detected between groups. For many disease-specific comparisons, ordering and adherence to quality of care indicators was low in both groups.

**Conclusions:**

We were unable to detect differences in quality of care between bedspaced and nonbedspaced patients. As high patient volumes and hospital overcrowding remains, bedspacing will likely continue. More research is required in order to determine if quality of care is compromised by this ongoing practice.

## Introduction

Overcrowding is an ongoing concern across hospitals [1]–[4]. When the number of patients requiring admission exceeds the number of available inpatient beds for a given hospital service, patients are often “bedspaced” to another inpatient unit, rather than wait for admission and occupy a much-needed Emergency Department (ED) bed. For example, patients admitted to the general internal medicine (GIM) service would be physically placed on a non-GIM ward. The “bedspaced” wards are typically located in geographically separate areas of the hospital from the GIM ward. While medical care is the responsibility of the admitting service, allied health services (e.g. nursing and physiotherapy) are typically administered by the “host” service.

A shortage of inpatient beds is a contributing factor in ED overcrowding, and when ED volumes are high, the GIM service often serves as a “safety net”, admitting greater proportions of patients [1]–[3]. While bedspacing is a routine occurrence in many hospitals, it is unclear whether this practice compromises patient care. Evidence from similar patient populations who are physically separated from the medical team has shown that clinician-patient barriers can result in poorer quality of care [5]–[10]. As care needs may differ between GIM and non-GIM patients, non-GIM “host” services may have less experience with GIM patients, and ultimately, bedspaced patients may have compromised quality of care (for example, with poorer nursing adherence to quality measures) [11], [12]. Moreover, coordination of allied health care may be affected, including timely access to pharmacy medication reconciliation and assessment by physiotherapy, which may impact length of hospitalization. Therefore we hypothesized that bedspacing represents a physical clinician-patient barrier, resulting in longer hospital stays and increased rates of return to ED after discharge. We used explicit general and disease specific process measures and 30-day patient outcomes to determine whether bedspacing affects quality of care in hospitalized GIM inpatients.

## Methods

Approval from the St. Michael's Hospital Research Ethics Board was sought prior to the study and obtained. As this was a retrospective chart review of GIM admissions with all eligible charts de-identified for blinded chart review, participant consent forms were not required.

### Data Sources and Construction

We used hospital databases to identify consecutive GIM admissions for congestive heart failure (CHF), chronic obstructive pulmonary disease (COPD) and pneumonia from May 2007 to March 2011. This data was further sorted into admission date, admission physician, and hospital bedspace location in order to obtain matched pairs for each diagnosis (i.e. one patient remaining on the GIM wards and the other patient bedspaced off the GIM wards). Chart review of these selected admissions was conducted electronically by the primary author (JL) by reviewing scanned patient charts in their entirety, in order to generate a database of the patient demographics and outcomes of interest. Data sources included medical notes, nursing vital sign records, Emergency Department facesheets, written orders and discharge summaries via online retrospective chart review of scanned patient charts as described previously [13]–[15]. This study was conducted prior to the hospital implementation of computerized order entry and diagnosis-specific order sets.

### Study Setting, population and the matching process

St. Michael's Hospital is a tertiary-care, 513-bed, downtown teaching hospital affiliated with the University of Toronto. The 64-bed GIM service is comprised of four clinical teams of a staff physician and 3–5 housestaff (a senior GIM resident, first-year residents and medical students) who are responsible for writing all admission and care orders.

**Figure 1:**
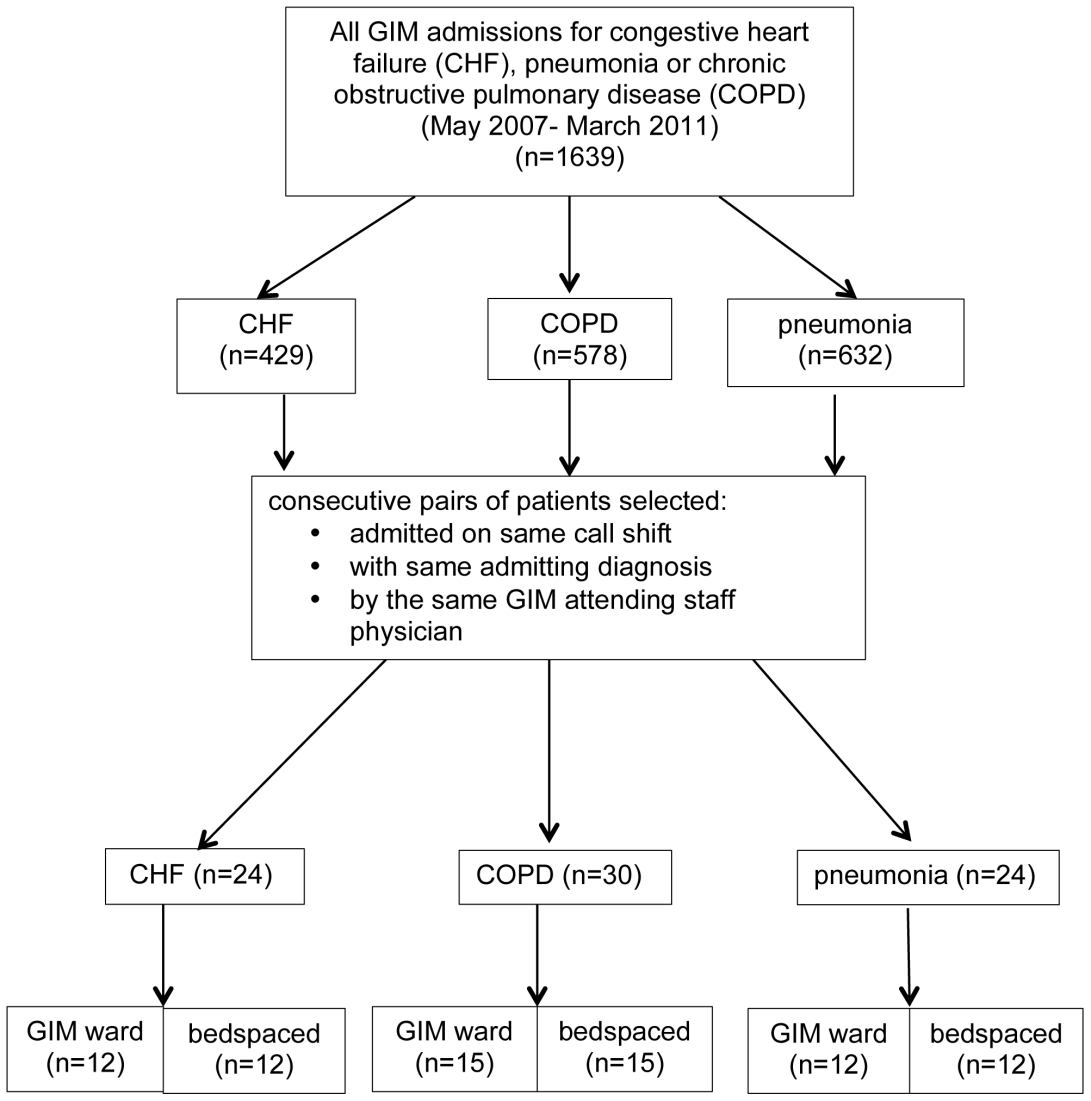
Admitting diagnosis and bedspace status of consecutive admissions to General Internal Medicine (GIM) (May 2007–March 2011).

We followed a matched cohort design, where each “exposed” (bedspaced) patient was matched with an “unexposed” (nonbedspaced) patient. Each pair was admitted with the same admitting diagnosis (CHF, COPD, or pneumonia), during the same call shift under the same attending physician, in order to control for temporal differences as well as physician differences in clinical management (Figure 1). These diagnoses were chosen as they are among the top five GIM admission diagnoses at the hospital.

### Bedspacing

We defined a patient as “bedspaced” when they were admitted to the GIM service, but physically located to an inpatient unit other than the GIM wards. At this hospital, non-GIM wards included both surgical wards (i.e. vascular surgery, orthopedic surgery, neurosurgery, urology, plastic surgery, general surgery) and nonsurgical wards (human immunodeficiency virus, hematology/oncology, respirology, cardiology, nephrology, and neurology) inpatient units. Several non-GIM inpatient wards were a mix of surgical patients plus nonmedical specialty patients (e.g., neurosurgery plus neurology patients) or surgical patients plus subspecialty medicine patient (e.g. urology plus nephrology patients). We considered subspecialty medical wards such as cardiology, respirology, human immunodeficiency virus, hematology/oncology and gasteroenterology as bedspaced off the GIM wards, as criteria for admission to these wards under a medical subspecialist are highly specialized and often unique to that specialty (For example, St. Michael's Hospital has a large cystic fibrosis program; therefore, many of the admitted respirology patients are cystic fibrosis patients).

### Point-of-care process for bedspacing

At this hospital, when the number of GIM patients requiring admission exceeds bedspace capacity, it is the responsibility of Patient Flow Managers and Admitting staff to secure offservice patient beds. The decision regarding where to bedspace a GIM patient is driven by: (a) which services have available beds (with nonsurgical wards given priority over surgical wards), and (b) length of time the patient has been in the Emergency Department since admission. No formal consideration is given to patient comorbidities, level of acuity, proposed ELOS, or discharge planning. Isolation status is the only practical issue that is formally considered and, if possible, GIM patients requiring isolation for infection control are not bedspaced.

### Primary outcome

The primary outcome was an adjusted hospital length of stay measure known as the estimated length of stay (ELOS). The ELOS is a computed estimated value defined by the Canadian Institute for Health Information that reflects the expected length of hospitalization, adjusted for age, diagnosis, medical comorbidities and in-hospital resource intensity weights for Canadian acute care hospitals. The ELOS was chosen over the actual length of stay specifically because this value attempts to correct for potential sources of confound. The ELOS measures are derived from the current 2 years of patient LOS data based on all acute care hospitals in Canada [16]. In order to facilitate comparisons between patients with respect to whether or not they remained in hospital longer than anticipated, even after adjusting for age, diagnosis and medical comorbidities, the percentage ELOS (% ELOS) was calculated for each patient by dividing the actual hospital length of stay by the ELOS.

### Secondary Outcome

The secondary outcome included the rate at which discharged patients returned to the same hospital's Emergency Department (ED) within 30 days of discharge. This measure was selected as previous work has shown that approximately 70% of patients who represent to the ED within 30 days of discharge return to the same hospital [14], [17]–[18]. We noted which patients returned to the ED, and followed whether they were discharged home or readmitted to another service. We also reviewed these patient charts in order to ascertain the reason for the representation. The secondary outcome was a composite of this 30-day representation rate and both general and disease specific process of care measures.

### General process of care measures

We evaluated ten general quality measures: (1) nursing adherence to vital sign measurement; (2) frequency of respiratory rate documentation as 20 breaths per minute; (3) frequency of missing daily medical progress notes; (4) frequency of documented physical exam findings; (5) frequency of progress notes charted before noon; (6) early documentation of pharmacy medication reconciliation; (7) early documentation of patient's resuscitation status; (8) early ordering of deep vein thrombosis (DVT) prophylaxis (or rationale as to why not); (9) early ordering of physical therapy; and (10) early assessment by physical therapy. We defined “early” as within twenty-four hours of admission or ordering. These quality measures were selected to reflect thoroughness of care, for their appropriateness for all diagnoses, and for their use in previous quality of care research [19]–[22].

### Disease specific process of care measures

For CHF, we assessed: (1) whether daily weights, fluid restriction, accurate fluid balances, a low-salt diet and daily weights were ordered and recorded; (2) whether more than three sets of vitals were ordered on the second day of admission; (3) the percentage of daily progress notes with volume status charted; (4) whether left ventricular (LV) function was evaluated or arranged prior to discharge; (5) whether an angiotensin-converting enzyme (ACE) inhibitor, beta-blocker, or warfarin were prescribed if indicated (or documented as to why not) [23]–[28]. For COPD, care measures included whether: (1) an arterial blood gas (ABG) was measured on admission if oxygen saturation was less than 90%; (2) oxygenation parameters were ordered to 88–92% in the setting of hypercapnia; (3) antibiotics were ordered if indicated; (4) beta blocker doses were not started or increased; (5) respiratory peak flows were ordered and measured; (6) smoking cessation discussions were documented if applicable [22], [29]–[31]. For pneumonia, we evaluated whether: (1) antibiotics were administered within 6 hours of ED triage; (2) oxygen saturation was documented on admission; (3) two sets of blood cultures were drawn before antibiotic administration; (4) the pneumococcal or influenza vaccine were given if indicated; and (5) smoking cessation discussions were documented if indicated [32]–[34].

### Patient Dissatisfaction

We intended to evaluate whether bedspacing affected the frequency of formal complaints filed by patients [19]. However, no complaints were filed with the Patient Affairs Office for any patients in this study.

### Repatriation

If bedspaced, when GIM ward beds eventually became available, patients were subsequently transferred to the GIM wards. We referred to this process as “repatriation”, and noted if and when repatriation occurred for every bedspaced patient.

### Statistics

For binary outcomes, we estimated matched risk ratios (RR) and 95% confidence intervals (CI) as an extension of Mantel–Haenszel methods implemented in Stata version 11's command csmatch (StataCorp. 2009. *Stata Statistical Software: Release 11*. College Station, TX: StataCorp LP). Matching was broken when pairs were incomplete; i.e., if the outcome was not applicable for both patients in each pair. In these (infrequent) instances, unmatched risk ratios were estimated. When outcomes were not binary, we applied a clinically relevant proportion to both groups; e.g., for vital sign monitoring adherence, we considered 75% adherence as clinically meaningful. ELOS and per cent ELOS were compared between groups using the Wilcoxon signed-rank test.

## Results

There were 1639 consecutive GIM admissions for CHF, COPD or pneumonia from May 2007 to March 2011. Of these, 39 bedspaced patients were matched with 39 GIM ward patients with the same admission date, attending physician and admission diagnosis (Figure 1). Baseline characteristics of both groups were similar (Table 1). Mean age was 67.5 years. Nearly half of patients were female (41% bedspaced vs. 46% GIM), and approximately half were isolated for infection control during admission (51.3% bedspaced vs. 43.6% GIM). About half of all patients (51.3%) were admitted on the weekend. The majority of bedspaced patients went to subspecialty medicine wards rather than surgical wards (53.8% vs. 30.8%), and five patients (12.8%) of patients went to a mixed surgical and medical subspecialty ward. One-third of bedspaced patients (36%) were ultimately repatriated—on average, 2.9 days after admission. With respect to discharge, a greater proportion of bedspaced patients were discharged home compared with GIM ward patients (71.8% vs. 43.6%), as opposed to alternate discharge destinations such as rehabilitation hospitals or long term care centres.

**Table 1:** Patient demographics of General Internal Medicine (GIM) ward and bedspaced patients.

		General Internal Medicine (GIM) ward (n = 39)	Bedspaced offservice (n = 39)
Mean age and standard deviation (SD)		66.9 (15.7)*	68.4 (17.0)*
	Female	18 (46.1%)	16 (41.0%)
	English-speaking primarily	33 (84.6%)	29 (74.4%)
Admitting diagnosis:			
	Congestive heart failure (CHF)	12 (30.1%)	12 (30.1%)
	Chronic Obstructive Pulmonary Disease (COPD)	15 (38.5%)	15 (38.5%)
	Pneumonia	12 (30.1%)	12 (30.1%)
Isolation at any point in admission		17 (43.6%)	20 (51.3%)
Admitted on weekend		20 (51.3%)	20 (51.3%)
Discharged to	Died	1 (2.5%)	0
	Home	17 (43.6%)	28 (71.8%)
	Rehab	5 (12%)	3 (7.7%)
	Nursing home	7 (18.0%)	2 (5.1%)
	Shelter/No fixed address	4 (10.3%)	6 (15.4%)
	Jail	1 (2.6%)	0
	Transfer to another hospital	1 (2.6%)	0
	Transfer to another service	1 (2.6%)	0
	Left against medical advice	2 (5.1%)	0
Ever smoker		21 (63.6%)	24 (66.7%)
Alcohol use		12 (40.0%)	14 (45.2%)
Intravenous drug use		1 (5.6%)	2 (8.7%)
No fixed address		3 (7.7%)	6 (15.4%)
Bedspace location	Surgical ward	-	12 (30.8%)
	Subspecialty medicine ward	-	21 (53.8%)
	Mixed surgical/subspecialty medicine ward	-	5 (12.8%)
	Remained in Emergency Department	-	1 (2.6%)
Percentage repatriated to GIM ward		-	14 (36%)
Mean date of repatriation		-	1.3 days

*Standard deviation (SD)

Binary and categorical data are presented as n(%), and continuous variables as mean (SD). Proportions may not add to 100% due to rounding.

### Primary outcomes

Bedspaced patients had a similar length of hospital stay compared with GIM ward patients (4.9 vs. 6.0 days, median 5.0 vs. 4.0, p = 0.30). The mean calculated ELOS for bedspaced patients was 6.6 days, compared with 7.0 for GIM ward patients (median 6.0 and 6.0, p = 0.92). Similarly, bedspaced and GIM patients had similar mean percentage ELOS (75.8% of ELOS vs. 84.1%; p = 0.18) (Table 2.).

**Table 2:** Percentage of estimated length of stay (ELOS) and representation to hospital within 30 days of discharge.

	General Internal Medicine (GIM) wards (n = 39)	Bedspaced offservice (n = 39)	Test statistic
Length of stay (LOS)	6.02	4.85	p = 0.30^A^
			
Mean estimated LOS (ELOS)	7 (2.1)	6.6 (3.8)	p = 0.92^A^
			
Percentage (%) ELOS	84.1%	74.5%	p = 0.18^A^
Percentage (%) represented to Emergency Department or readmitted within 30 days	12 (30.8%)	8 (20.5%)	0.67^B^ (0.31–1.45)
Represented after x days (median)	8.0 (24.5)^C^	6.5 (10)^D^	p = 0.67^E^

A p value, two-sided Wilcoxon rank-sum test.

B risk ratio (95% CI).

C median.

D quartile range.

E p value, two-sided Wilcoxon rank-sum test (unmatched analysis).

### Representation to the Emergency Department within 30 days of discharge

One-fifth of bedspaced patients (20.5% or 8/39) as compared with one-third of GIM ward patients (30.8% or 12/39) represented to the ED within 30 days (RR 0.67; 95% CI 0.35–1.25). Median representation times showed that bedspaced patients returned to the ED at 6.5 days post discharge compared with 8.0 days if originally admitted to the GIM ward (p = 0.68) (Table 2). Approximately one-third of patients originally admitted to the GIM wards (30.8% or 12/39) returned to the ED within 30 days as compared to one-fifth of bedspaced patients (20.5% or 8/39). Of the GIM group, over half (7/12 or 58%) were readmitted to hospital, all under GIM as compared with half of bedspaced patients (4/8 or 50%), although only one quarter (2/8 or 25%) of these patients were readmitted to GIM. Notably, of the GIM subgroup, half of GIM repeat patients (6/12 or 50%) were given exactly same diagnosis as their previous admission (i.e. CHF for CHF), and a further quarter of patients (3/12 or 25%) had provisional diagnoses that were easily extrapolatable to their original admission diagnoses (i.e. “dyspnea” in a COPD admission, “chest discomfort” in a pneumonia patient). For bedspaced patients, only 1/8 had the same diagnosis as their original admission (Table 3).

**Table 3:** Representation to Emergency Department (ED) and disposition of bedspaced and GIM ward patients within 30 days of initial discharge.

Diagnosis	Bedspaced status	Description	Disposition
pneumonia	GIM	Chest discomfort	Readmitted to GIM
COPD	GIM	COPD	Readmitted to GIM
CHF	GIM	CHF	Readmitted to GIM
pneumonia	GIM	Unclear	Represented to ER and discharged home
COPD	GIM	Upper gastrointestinal bleed	Readmitted to GIM
COPD	GIM	Dyspnea	Represented to ER and discharged home
CHF	GIM	CHF	Readmitted to GIM
COPD	GIM	Unclear	Represented to ER and discharged home
COPD	GIM	COPD	Readmitted to GIM
COPD	GIM	COPD	Readmitted to GIM
COPD	GIM	Chest pain NYD	Represented to ER and discharged home
COPD	GIM	COPD	Represented to ER and discharged home
CHF	Offservice	Bowel Obstruction	Admitted to General Surgery
COPD	Offservice	Leg swelling NYD	Represented to ER and discharged home
COPD	Offservice	Unclear - referred to GI	Represented to ER and discharged home
CHF	Offservice	Foot fracture	Represented to ER and discharged home
COPD	Offservice	Dyspnea	Readmitted to GIM
CHF	Offservice	Epistaxis	Represented to ER and discharged home
COPD	Offservice	COPD	Admitted to Respirology
COPD	Offservice	Diarrhea	Readmitted to GIM

### General Process of Care Measures

Both groups had similar adherence to vital sign monitoring (mean 94.4% bedspaced vs. 95% GIM; p = 0.85) and had documented respiratory rates as 20 breaths/minute at similar frequencies (37% bedspaced vs. 44% GIM; p = 0.31). If bedspaced, nurses were as likely to have greater than 75% adherence to vital sign monitoring (RR 1.0 (95% CI 0.77–1.29) as for nonbedspaced patients. The likelihood of charting respiratory rates of 20 breaths/minute more than 50% the time did not differ between groups (RR 0.62; 95% CI 0.35–1.09). Both groups were equally likely to have missing daily progress notes (i.e., greater than 25%) (RR 1.5; 95% CI 0.77–2.92), adequate charting of physical exam findings (more than 75% of all notes) (RR 1.09; 95% CI 0.77–1.54), morning progress notes (greater than 50% of all days) (RR 0.67; 95% CI 0.34–1.30), and an early attending staff note (RR 0.91; 95% CI 0.73–1.13). Bedspaced patients were as likely to have early DVT prophylaxis prescription (RR 0.90; 95% CI 0.63–1.26), early documentation of resuscitation status, (RR 0.86; 95% CI 0.64–1.15), early physical therapy consultation (RR 0.84; 95% CI 0.51–1.38), and early physical therapy assessment (RR 1.15; 95% CI 0.75–1.77) (Table 4).

**Table 4:** General process of care measures for General Internal Medicine (GIM) ward vs. bedspaced patients.

General process of care measures	General Internal Medicine (GIM) ward	Bedspaced offservice	risk ratio (RR); 95% CI matched analysis (unless specified)
Total vital signs expected (mean)	18.0	14.1	N/A
Total vital signs recorded (mean)	14.0	12.7	N/A
Adherence to ordered vitals, %	94%	95%	p = 0.97^A^
Adherence to ordered vitals (>75%)	N/A	N/A	1.0 (0.77–1.29)
Vital signs with respiratory rate (RR) 20/min, %	44%	37%	p = 0.31^A^
RR 20/min (>50% of all vitals)	N/A	N/A	0.62 (0.35–1.09)
Admission days with missing medical progress note, (>25%)	17.9%	20.0%	1.5 (0.77–2.92)
Days with progress note with physical exam findings charted (>75%)	72.1%	75.3%	1.09 (0.77–1.54)
Days with progress note clearly documented before noon, (>50%)	38.4%	30.3%	0.67 (0.34–1.30)
Staff note within 24 hrs of admission	33/39 (84.6%)	30/39 (76.9%)	0.91 (0.73–1.13)
Code status documented in <24 h	29/39 (74.4%)	25/39 (64.1%)	0.86 (0.61–1.16)
DVT prophylaxis within 24 h	25/39 (64.1%)	22/39 (57.9%)	0.88 (0.61–1.26)
PT ordered within 24 h	19/39 (48.7%)	17/39 (43.6%)	0.84 (0.51–1.38)
PT assessed within 24 h	13/15 (86.7%)	13/16 (81.3%)	1.15 (0.75–1.77)^*^

A p value, students' t-test; *unmatched analysis used for risk ratio.

N/A  =  statistical analysis or calculation was not applicable or appropriate.

General care measures differed across categories of the three disease groups were also examined. Overall, significant differences were not detected between bedspaced patients vs. nonbedspaced patients when subcategorized into their admitting diagnoses. Groups had similar adherence to vital sign monitoring, documentation of medical notes, DVT prophylaxis and code status, as well as early attending physician notes. Mean percentage of ELOS for admitting diagnoses of CHF and pneumonia were slightly longer for GIM ward patients as compared to bedspaced patients; however, these results were not statistically significant (Tables S1–S3).

### Disease Specific Process of Care Measures

Both bedspaced and GIM ward patients were as likely to have 3 vital sign measurements charted on day two of admission (RR 1.37; 95% CI 0.78–2.42), and as likely to be prescribed an ACE inhibitor (RR 0.89; 95% CI 0.53–1.49), a beta-blocker (RR 1.0; 95% CI 0.78–1.29), and warfarin if indicated (RR1.0; 95% CI 0.78–1.29), and as likely to undergo LV function evaluation (RR 0.91; 95% CI 0.66–1.26). Both groups were as likely to be ordered daily weights, fluid restriction, accurate fluid balances and a low salt diet (Table 5); however, these measures were so infrequently ordered (e.g. for daily weights, 4/12 in each group) that meaningful statistical comparisons with respect to adherence were not completed (Table 5).

**Table 5:** Disease specific process of care measures for general internal medicine (GIM) vs. bedspaced patients.

	General Internal Medicine (GIM) ward	Bedspaced offservice	Test statistic (risk ratio; matched analysis unless *)
**Congestive Heart Failure (CHF) (n = 24)**			
Daily weights ordered	4/12(33.3%)	4/12 (33.3%)	No difference
Fluid restriction ordered	2/12 (16.7%)	5/12 (41.6%)	2.5 (0.63–9.99)
Fluid balance ordered	5/12 (41.7%)	4/12 (33.3%)	0.80 (0.25–2.55)
Low salt diet ordered	6/12 (50.0%)	3/12 (25.0%)	0.50 (0.17–1.40)
>3 vitals on day 2 of admission	7/12 (58.3%)	8/10 (80%)	1.37 (0.78–2.42)*
Progress notes with volume status (%)	79.2%	79.6%	No difference
Left ventricular (LV) function evaluation completed or arranged prior to discharge	11/12(91.7%)	10/12 (83.3%)	0.91 (0.66–1.26)
Angiotensin-converting enzyme (ACE) inhibitor prescribed at discharge	9/12 (66.7%)	8/12 (75.0%)	0.89 (0.53–1.49)
Beta blocker (BB) prescribed at discharge	11/12 (92%)	11/12 (92%)	1.0 (0.78–1.29)
Warfarin prescribed at discharge if atrial fibrillation	11/12 (92%)	11/12 (92%)	1.0 (0.78–1.29)
**Chronic Obstructive Pulmonary Disease (COPD) (n = 30)**			
ABG on admission if oxygen saturation noted to be<90%	4/5 (80%)	5/8 (62.5%)	0.99 (0.74–1.32)*
Antibiotics if appropriate	9/9 (100%)	12/12 (100%)	No difference
Oxygen parameters set to 88–92% saturation if pH<7.45	14/15 (93.3%)	9/15 (60.0%)	0.64 (0.44–0.95)
New/increased BB dose NOT ordered	11/12(93.3%)	11/12 (93.3%)	No difference
Respiratory peak flow ordered	3/15 (20%)	1/15 (6.7%)	0.3 (0.03–2.85)
Smoking cessation discussed	4/15 (26.7%)	4/15 (26.7%)	No difference
**Pneumonia (n = 24)**			
Antibiotics within 6 hours of triage	7/12 (58.3%)	8/12 (66.7%)	1.14 (0.61–2.13)
Oxygen saturation documented	12/12 (100%)	12/12 (100%)	No difference
2 blood cultures prior to antibiotics	10/12 (83.3%)	9/12 (75.0%)	0.90 (0.73–1.11)
Appropriate antibiotics	1/1 (100%)	4/4 (100%)	No difference
Pneumococcus vaccine given if appropriate (or reasons as to why not)	5/12 (41.7%)	8/12 (66.7%)	1.60 (0.73–3.49)
Influenza vaccine if appropriate (or reasons as to why not)	6/12 (50.0%)	5/12 (41.7%)	0.83 (0.35–2.0)
Smoking cessation discussed if applicable	0/7 (0%)	0/7 (0%)	No difference

*unmatched risk ratio reported because matching process was broken.

For COPD, bedspaced patients were as likely as GIM ward patients to have an admission arterial blood gas (ABG) measurement for oxygen saturations of less than 90% (risk ratio 0.99; CI 0.74–1.32), and were less likely to be ordered oxygen saturation parameters of 88–92% if hypercapneic (risk ratio 0.64; 95% CI 0.44–0.95). All patients were equally likely to be ordered appropriate antibiotics (100% both groups), have charted smoking cessation discussions (26.7% both groups), and not have beta-blocker dosages increased (93% both groups). Several disease specific measurements, such as peak flows, were ordered so infrequently (e.g. 3/15 of GIM-ward patients) that analysis of adherence was not performed.

All patients with pneumonia had oxygen saturations charted by the admitting physician, and none had documented smoking cessation education. Groups did not differ in likelihood of receiving antibiotics within 6 hours of triage (RR 1.14; 95% CI 0.61–2.13), and to have blood cultures drawn twice prior to antibiotic administration (RR 0.90; 95% CI 0.73–1.11). If candidates, both groups were as likely to receive the pneumococcal (1.60; 95% CI 0.80–3.2) and the influenza vaccine (0.83; 95% CI 0.37–1.85).

## Discussion

We compared consecutive admissions for congestive heart failure, chronic obstructive pulmonary disease and pneumonia and matched for diagnosis, call period and medical team to determine whether bedspacing compromises quality of care. We found that bedspacing was unrelated to length of stay, even when corrected for diagnosis and medical comorbidities. Groups had similar adherence to both general and disease specific process of care measures. We were not able to find evidence that there was poorer adherence to quality of care indicators if bedspaced; however, this may reflect the small sample sizes with limited statistical power. In particular, for disease specific quality of care measures, frequency of ordering these indicators was so low in both groups that further analysis was precluded. It is possible that several of our parameters may have achieved statistical significance with increased sample sizes.

A strength of our study is how stringently our matching process sought to control for physician-specific, diagnosis-specific and temporal differences in patient care, thereby best isolating differences associated with the practice of bedspacing. As well, our consecutive cohort included both general and diagnosis-specific measures to assess for differences that may have been subtle. Further, we evaluated patients with differing diagnoses. In addition, to our knowledge, this study is among the first to address the practice of bedspacing.

The hypothesis that patient-physician barriers affect care is not new. However, as there is a paucity of literature on bedspacing itself, we feel that relevant inferences may be drawn from the Emergency Department (ED) overcrowding literature. Patients who remain in the ED after admission, but do not yet have an inpatient bed, have been referred to as “boarders.” Evidence suggests that boarders have increased lengths of stay, delays in home medication administration, and overall delays in medical care [5]-[9]. One study demonstrated an association with increased in-hospital mortality rates [10]. One purported hypothesis for poor boarder quality outcomes is reduced clinician-patient contact imposed by boarding, ultimately leading to delayed therapeutic interventions and poorer care [8]. Another related area would be quality of care differences related to patients placed under infection control isolation. Indeed, patients isolated for infection control are twice as likely as controls to experience in-hospital adverse events, more likely to have missing physician progress notes, and more likely to have poor adherence to vital sign measurements [19]. While we did not find bedspacing affected the frequency of charted progress notes or timeliness of a staff physician note, this may not be a direct surrogate for quality of care.

That nearly 50% of our study patients were admitted on the weekend is not surprising, as hospital overcrowding is the impetus for bedspacing in the first place. On weekends, there may be fewer medical personnel available for clinical care and discharge planning; as fewer patients are discharged from the GIM service, the need for bedspacing thus increases. For certain diagnoses, admission on a weekend has been associated with significantly higher in-hospital mortality rates than were weekday admissions [35]. Whether bedspacing affects outcomes such as mortality was outside the scope of this study; however, weekends may be a vulnerable time for bedspacing to occur, based on hospital occupancy.

We acknowledge that this study has several limitations. First, we were unable to control explicitly for illness severity. In our matching process, more acutely ill patients may have been preferentially admitted to the GIM ward, reflecting a potential source of selection bias. It is interesting that compared with GIM patients, a greater proportion of bedspaced patients were discharged home—and a smaller proportion of patients were discharged to a rehabilitation hospital or nursing home, which could relate to lower acuity of illness among bedspaced patients. Moreover, that a greater proportion of patients who were originally admitted to GIM returned to hospital and were readmitted to the same service with a similar diagnosis is an interesting finding, and may reflect a higher level of medical acuity in these patients. In some instances, exposure to bedspacing was abbreviated. Repatriation occurred in approximately one third of bedspaced patients, and, on average, occurred on their third day of admission, further hindering our ability to elucidate any potential differences between groups. A final limitation was how infrequently certain measures occurred. For example, only one third of CHF patients were ordered daily weights. In such cases, adherence was so infrequent that we could not detect or claim differences between groups. However, for many of these parameters, statistical significance may have been achieved simply by increasing the sample size. This was likely a consequence of our stringent matching process.) The possibility remains that our selected process of care measures were not sufficiently sensitive to detect differences between groups. However, these measures have been used in other studies [19]–[34], several of which have demonstrated differences in outcomes such as mortality and reduced return to hospital [23]–[24], [27]. In addition, we may have been overly stringent in our attempt to control for confound. Moreover, confidence intervals were wide and we could not exclude large and potentially clinically significant risk ratios between groups.

With ongoing high patient volumes and hospital overcrowding, bedspacing will likely continue. While we were unable to detect differences in care between bedspaced and nonbedspaced patients, more research is required to determine whether or not quality of care is compromised by this ongoing practice. We believe our research highlights a gap in current knowledge regarding the quality implications of existing hospital practices. We recommend that hospitals examine current data and patient outcomes with respect to bedspacing in order to discern any potential existing differences in quality of care. Until then, hospitals must be aware of this possibility and continue to ensure that patients receive the same high standard of medical care regardless of their location in the hospital.

## Supporting Information

Table S1 **General process of care measures, percentage of estimated length of stay (ELOS) and representation to hospital within 30 days of discharge: congestive heart failure (CHF).**
(DOCX)

Table S2 **General process of care measures, percentage of estimated length of stay (ELOS) and representation to hospital within 30 days of discharge: Chronic Obstructive Pulmonary Disease (COPD).**
(DOCX)

Table S3 **General process of care measures, percentage of estimated length of stay (ELOS) and representation to hospital within 30 days of discharge: pneumonia.**
(DOCX)
