# Supplementary material for: Quality of Care of Hospitalized Internal Medicine Patients Bedspaced to Non-Internal Medicine Inpatient Units
Source: PLoS One. 2014 Sep 3;9(9):e106763. doi: 10.1371/journal.pone.0106763 (PMC4153721; doi:10.1371/journal.pone.0106763)
Supplement: Table S1 — General process of care measures, percentage of estimated length of stay (ELOS) and representation to hospital within 30 days of discharge: congestive heart failure (CHF). (DOCX) [file pone.0106763.s001.docx]

**Supplementary Table 1. General process of care measures, percentage of estimated length of stay (ELOS) and representation to hospital within 30 days of discharge: congestive heart failure (CHF)**

|  | **General Internal Medicine (GIM) ward** | **Bedspaced**  **off service** | **Test statistic (risk ratio; matched analysis unless *)** |
| --- | --- | --- | --- |
| Total vital signs expected (mean) | 9.3 | 21.7 | N/A |
|  |  |  |  |
| Total vital signs recorded (mean) | 9.7 | 19.7 | N/A |
|  |  |  |  |
| Adherence to ordered vitals, % (SD) | 109.49 (38.82) | 90.08 (47.55) | p=0.29^A^ |
| Adherence to ordered vitals (>75%) | 10/12 | 9/12 | 0.90 (0.57-1.43) |
|  |  |  |  |
| Vital signs with respiratory rate (RR) 20/min, % (SD) | 42.71(21.70) | 25.13(30.23) | p=0.12^A^ |
| RR 20/min (>50% of all vitals) | 6/12 | 2/12 | 0.33 (0.07-1.65) |
|  |  |  |  |
| Admission days with missing medical progress note, (>25%) | 6/12 | 3/12 | 2.0 (0.71-5.62) |
| Days with progress note with physical exam findings charted (>75%) | 9/12 | 8/12 | 0.89 (0.53-1.49) |
|  |  |  |  |
| Days with progress note clearly documented before noon, (>50%) | 5/12 | 3/12 | 0.60 (0.17-2.07) |
|  |  |  |  |
| Staff note within 24hours of admission | 11/12 | 10/12 | 0.91 (0.66-1.26) |
|  |  |  |  |
| Code status documented in <24h | 8/12 | 10/12 | 1.25 (0.81-1.94) |
|  |  |  |  |
| DVT prophylaxis within 24h | 8/12 | 6/12 | 0.75(0.38-1.50) |
|  |  |  |  |
| PT ordered within 24h | 6/12 | 6/12 | 1.0 (0.45-2.22) |
|  |  |  |  |
| Repatriated | N/A | 4/12 | N/A |
| Mean date of repatriation, days | N/A | 3.0 days | N/A |
|  |  |  |  |
| Length of stay (LOS) | 8.5 (5.74) | 4.17 (1.99) | p=0.07^B^ |
|  |  |  |  |
| Mean estimated LOS (ELOS) | 8.67 (5.82) | 6.14 (1.47) | p=0.30^B^ |
|  |  |  |  |
| Percentage (%) ELOS | 100 (46) | 71 (39) | p=0.08^B^ |
|  |  |  |  |
| Representation to ER within 30 days | 2/12 | 3/12 | 1.5 (0.35-6.0) |
|  |  |  |  |
| Representation after x days, mean (median) | 9.5 (9.5) | 5 (5) | p=0.77^B^ |

^A^ p value, t-test ^B^ p value, two-sided Wilcoxon rank-sum test (unmatched analysis)
