# Supplementary material for: Quality of Care of Hospitalized Internal Medicine Patients Bedspaced to Non-Internal Medicine Inpatient Units
Source: PLoS One. 2014 Sep 3;9(9):e106763. doi: 10.1371/journal.pone.0106763 (PMC4153721; doi:10.1371/journal.pone.0106763)
Supplement: Table S2 — General process of care measures, percentage of estimated length of stay (ELOS) and representation to hospital within 30 days of discharge: Chronic Obstructive Pulmonary Disease (COPD). (DOCX) [file pone.0106763.s002.docx]

|  | **General Internal Medicine (GIM) ward** | **Bedspaced**  **off service** | **Test statistic (risk ratio; matched analysis unless *)** |
| --- | --- | --- | --- |
| Total vital signs expected (mean) | 11.93 | 15.00 | N/A |
|  |  |  |  |
| Total vital signs recorded (mean) | 9.27 | 15.00 | N/A |
|  |  |  |  |
| Adherence to ordered vitals, % (SD) | 85.47 (36.64) | 90.74 (36.21) | p=0.69^A^ |
| Adherence to ordered vitals (>75%) | 11/15 | 11/15 | 1.0 (0.65-1.55) |
|  |  |  |  |
| Vital signs with respiratory rate (RR) 20/min, % (SD) | 34.12 (31.15) | 46.17 (36.76) | p=0.35^A^ |
| RR 20/min (>50% of all vitals) | 6/15 | 4/14 | 1.4 (0.5-3.94)* |
|  |  |  |  |
| Admission days with missing medical progress note, (>25%) | 3/15 | 2/15 | 0.67 (0.11-3.99) |
| Days with progress note with physical exam findings charted (>75%) | 10/15 | 11/15 | 1.10 (0.67-1.80) |
|  |  |  |  |
| Days with progress note clearly documented before noon, (>50%) | 6/15 | 4/15 | 0.67 (0.30-1.48) |
|  |  |  |  |
| Staff note within 24hours of admission | 13/15 | 12/15 | 0.92 (0.70-1.21) |
|  |  |  |  |
| Code status documented in <24h | 12/15 | 10/15 | 0.83 (0.58-1.19) |
|  |  |  |  |
| DVT prophylaxis within 24h | 9/15 | 11/15 | 1.22 (0.66-2.28) |
|  |  |  |  |
| PT ordered within 24h | 7/15 | 8/15 | 1.14 (0.57-2.29) |
|  |  |  |  |
| PT assessed within 24h | 3/7 | 2/8 | 0.67 (0.30-1.48)* |
| Repatriated | N/A | 8/15 | N/A |
| Mean date of repatriation, days | N/A | 2.63 days | N/A |
|  |  |  |  |
| Length of stay (LOS), days (SD) | 3.87(2.53) | 5.07(2.58) | p=0.39^B^ |
|  |  |  |  |
| Mean estimated LOS (ELOS), days (SD) | 6.27(2.02) | 7.47(2.29) | p=0.15^B^ |
|  |  |  |  |
| Percentage (%) ELOS | 62(44) | 69(38) | p=0.85^B^ |
|  |  |  |  |
| Representation to ER within 30 days | 9/15 | 5/15 | 0.63(0.31-1.25) |
|  |  |  |  |
| Representation after x days, mean (median) | 6.75 (8.0) | 18.0 (27.0) | p=0.20^B^ |

**Supplementary Table 2. General process of care measures, percentage of estimated length of stay (ELOS) and representation to hospital within 30 days of discharge: Chronic Obstructive Pulmonary Disease (COPD)**

^A^ p value, t-test ^B^ p value, two-sided Wilcoxon rank-sum test (unmatched analysis)
