# Supplementary material for: Quality of Care of Hospitalized Internal Medicine Patients Bedspaced to Non-Internal Medicine Inpatient Units
Source: PLoS One. 2014 Sep 3;9(9):e106763. doi: 10.1371/journal.pone.0106763 (PMC4153721; doi:10.1371/journal.pone.0106763)
Supplement: Table S3 — General process of care measures, percentage of estimated length of stay (ELOS) and representation to hospital within 30 days of discharge: pneumonia. (DOCX) [file pone.0106763.s003.docx]

**Supplementary Table 3. General process of care measures, percentage of estimated length of stay (ELOS) and representation to hospital within 30 days of discharge: pneumonia**

|  | **General Internal Medicine (GIM) ward** | **Bedspaced**  **off service** | **Test statistic (risk ratio; matched analysis unless *)** |
| --- | --- | --- | --- |
| Total vital signs expected (mean) | 22.08 | 17.75 | N/A |
|  |  |  |  |
| Total vital signs recorded (mean) | 14.75 | 13.00 | N/A |
|  |  |  |  |
| Adherence to ordered vitals, % | 90.47(32.43) | 102.12(34.68) | p=0.40^A^ |
| Adherence to ordered vitals (>75%) | 8/12 | 9/12 | 1.13(0.75-1.68) |
|  |  |  |  |
| Vital signs with respiratory rate (RR) 20/min, % (SD) | 57.82(26.90) | 37.46(32.33) | p=0.11^A^ |
| RR 20/min (>50% of all vitals) | 9/12 | 4/12 | 0.44(0.21-0.92) |
|  |  |  |  |
| Admission days with missing medical progress note, (>25%) | 4/12 | 7/12 | 1.75(0.92-3.32) |
| Days with progress note with physical exam findings charted (>75%) | 4/12 | 6/12 | 1.5(0.56-4.00) |
|  |  |  |  |
| Days with progress note clearly documented before noon, (>50%) | 4/12 | 3/12 | 0.75(0.21-2.66) |
|  |  |  |  |
| Staff note within 24hours of admission | 9/12 | 8/12 | 0.89(0.53-1.49) |
|  |  |  |  |
| Code status documented in <24h | 9/12 | 5/12 | 0.56(0.24-1.27) |
|  |  |  |  |
| DVT prophylaxis within 24h | 8/12 | 6/12 | 0.75(0.38-1.50) |
|  |  |  |  |
| PT ordered within 24h | 6/12 | 3/12 | 0.5(0.18-1.40) |
|  |  |  |  |
| Repatriated | N/A | 2/12 | N/A |
| Mean date of repatriation, days | N/A | 3.5 days | N/A |
|  |  |  |  |
| Length of stay (LOS), days (SD) | 6.25(5.64) | 5.25(4.61) | p=0.39^B^ |
|  |  |  |  |
| Mean estimated LOS (ELOS) days (SD) | 6.25(2.77) | 6.00(2.22) | p=0.94^B^ |
|  |  |  |  |
| Percentage (%) ELOS | 96(51) | 89(82) | p=0.33^B^ |
|  |  |  |  |
| Representation to ER within 30 days | 2/12 | 0/12 | N/A |
|  |  |  |  |
| Represented after x days mean, (median) | 12.5 days (12.5) | N/A | N/A |

^A^ p value, t-test ^B^ p value, two-sided Wilcoxon rank-sum test (unmatched analysis)
